# Plain Hints for Busy Mothers

**Published:** 1903-12

**Authors:** 


Plain Hints for Busy Mothers. By Marianna Wheeler, Superintendent of the Babies' Hospital, New York, and author of The Baby. E. B. Treat & Co., New York. 1903. (Price, 35 cents.)

The object of this monograph is to instruct and advise the mother of limited means, living in a large city, in the matters of bathing, clothing, and feeding the young infant, in ventilation, out-door life, and the treatment of simple ailments. It is written in a succinct style and covers the essential points aimed at. If it could reach the mothers for whom it is intended, it would do much good. District nurses and settlement workers will find it a good book to introduce to their patients and neighbors.

